# Investigation of role of CpG methylation in some epithelial mesenchymal transition gene in a chemoresistant ovarian cancer cell line

**DOI:** 10.1038/s41598-022-11634-6

**Published:** 2022-05-06

**Authors:** Yaman Alghamian, Chadi Soukkarieh, Abdul Qader Abbady, Hossam Murad

**Affiliations:** 1 Department of Animal Biology, Faculty of Sciences, Damascus University, Damascus, Syria; 2 Human Genetics Division, Department of Molecular Biology and Biotechnology, Atomic Energy Commission of Syria (AECS), P.O. Box 6091, Damascus, Syria

**Keywords:** Cancer, Cell biology, Epigenetics, Gene expression, Gene regulation

## Abstract

Ovarian cancer is one of the lethal gynecologic cancers. Chemoresistance is an essential reason for treatment failure and high mortality. Emerging evidence connects epithelial-mesenchymal transition (EMT) like changes and acquisition of chemoresistance in cancers. Including EMT, DNA methylation influences cellular processes. Here, EMT-like changes were investigated in cisplatin-resistant A2780 ovarian cancer cells (A2780cis), wherein role of DNA methylation in some EMT genes regulations was studied. Cell viability assay was carried out to test the sensitivity of A2780, and A2780cis human cancer cell lines to cisplatin. Differential mRNA expression of EMT markers using qPCR was conducted to investigate EMT like changes. CpG methylation role in gene expression regulation was investigated by 5-azacytidine (5-aza) treatment. DNA methylation changes in EMT genes were identified using Methylscreen assay between A2780 and A2780cis cells. In order to evaluate if DNA methylation changes are causally underlying EMT, treatment with 5-aza followed by Cisplatin was done on A2780cis cells. Accordingly, morphological changes were studied under the microscope, whereas EMT marker’s gene expression changes were investigated using qPCR. In this respect, A2780cis cell line has maintained its cisplatin tolerance ability and exhibits phenotypic changes congruent with EMT. Methylscreen assay and qPCR study have revealed DNA hypermethylation in promoters of epithelial adhesion molecules *CDH1* and *EPCAM* in A2780cis compared to the cisplatin-sensitive parental cells. These changes were concomitant with gene expression down-regulation. DNA hypomethylation associated with transcription up-regulation of the mesenchymal marker *TWIST2* was observed in the resistant cells. Azacytidine treatment confirmed DNA methylation role in regulating gene expression of *CDH1*, *EPCAM* and *TWIST2* genes. A2780cis cell line undergoes EMT like changes, and EMT genes are regulated by DNA methylation. To that end, a better understanding of the molecular alterations that correlate with chemoresistance may lead to therapeutic benefits such as chemosensitivity restoration.

## Introduction

Ovarian cancer is one of the deadliest gynecologic cancers^1,2^. In 2021, 2% of all new cancer cases was estimated to be ovarian cancers^2^. Patients with ovarian cancer have a poor prognosis and high mortality rate due to its deceptive onset and lack of early diagnosis^3^. The low survival rate of ovarian cancer is due to metastasis and acquisition of chemoresistance^4^. Cisplatin (cis-diamminedichloroplatinum [II]) is a platinum compound widely used in solid tumors treatment many (including ovarian cancer)^5,6^. Cisplatin induces DNA lesions and activates several signaling pathways such as DNA repair mechanisms^7^. When DNA damage fails to be repaired, cells undergo apoptosis^8^. Cisplatin resistance is the main obstacle that limits the treatment effectiveness^9^. The mechanisms that underlie the platinum drug resistance are multifactorial. Several cellular processes are responsible for the resistant phenotype, such as drug influx or efflux alterations, DNA repair, cell cycle, and apoptosis^10,11^.

Recent evidence suggests that epithelial-mesenchymal transition (EMT) processes might play a role in ovarian cancer progression and chemoresistance development^12–17^. EMT is a cellular procedure where epithelial cells lose their cell–cell adhesion and cell polarity and gain a metastatic capability^18^. The molecular signatures of EMT are the loss of epithelial cell markers *CDH1* and the up-regulation of mesenchymal markers, which comprise EMT transcription factors *SNAIL* and *TWIST*^19,20^. Increased findings implicate EMT in promoting aggressiveness in ovarian cancer. Reports observed activation of EMT during the progression of ovarian cancer^21–23^. Expression signatures of enriched EMT genes have been observed and used to specify poor prognosis groups of ovarian cancer^23,24^. EMT induced an invasive phenotype in ovarian cancer cells through downregulation of CDH1 and up-regulation of CDH2 in Hey and OVCA433 cells^25^. EMT’s transcription factor SNAI1 induced EMT in ovarian cancer cells (SKOV3 cells) and enhanced the invasiveness^26^. Furthermore, EMT like changes have been reported in various chemoresistant cancers such as gastric cancer^27^, non-small cell lung cancer^28,29^, nasopharyngeal carcinoma cells^30^, breast cancer cells^31,32^, and ovarian cancer cells^16,17^. Many reports showed that ovarian cancer cells undergoing chronic exposure to cisplatin or paclitaxel generate cells that exhibit an EMT phenotype and molecular feature^17,33^. Additionally, multiple epigenetic events, such as DNA methylation, histone modifications, and non-coding RNA could contribute to the acquisition of chemoresistance^34–36^.

DNA methylation is the most well-known epigenetic mechanism that occurs at CpG islands influencing cellular processes by regulating gene expression^37^. Commonly, gene promoter hypermethylation is associated with reduced expression, while hypomethylation results in increased gene expression^38,39^. DNA methylation changes at CpG islands associated with transcriptional silencing have been described in cisplatin-resistant cancer cell lines^40–42^. For example, DNA methylation of several genes, including (*ARMCX2*, *COL1A1*, *MDK*, MEST, *MLH1*, *KLF4*, *ST3GAL5, SYNE1, CXCL8, HERC5, FOSL1,* and *ARRDC4*) was linked to ovarian cancer initiation and chemotherapy resistance^43,44^. A study in ovarian and breast cancer cell lines with doxorubicin tolerance described methylation changes in genes contributing to chemoresistance and identified hyper-methylation of *CDH1*, *BRCA1, SULF2* and *DNAJC15* genes, in addition to hypo-methylation of *APC, ABCB1* and *HIC1* genes^35^. Based on these considerations, the need to study the linkage between EMT and DNA methylation regulation has become a key to understanding the chemoresistant phenotype.

This study aims to determine if there are any significant differences in the expression of EMT gene regulators between two commercially-available ovarian cancer cell lines (A2780 and A2780cis), which differ in the degree of chemoresistance. This work also focuses on CpG methylation changes in these EMT genes.

## Results

### Cancer cells display different morphological shapes

**Figure 1:**
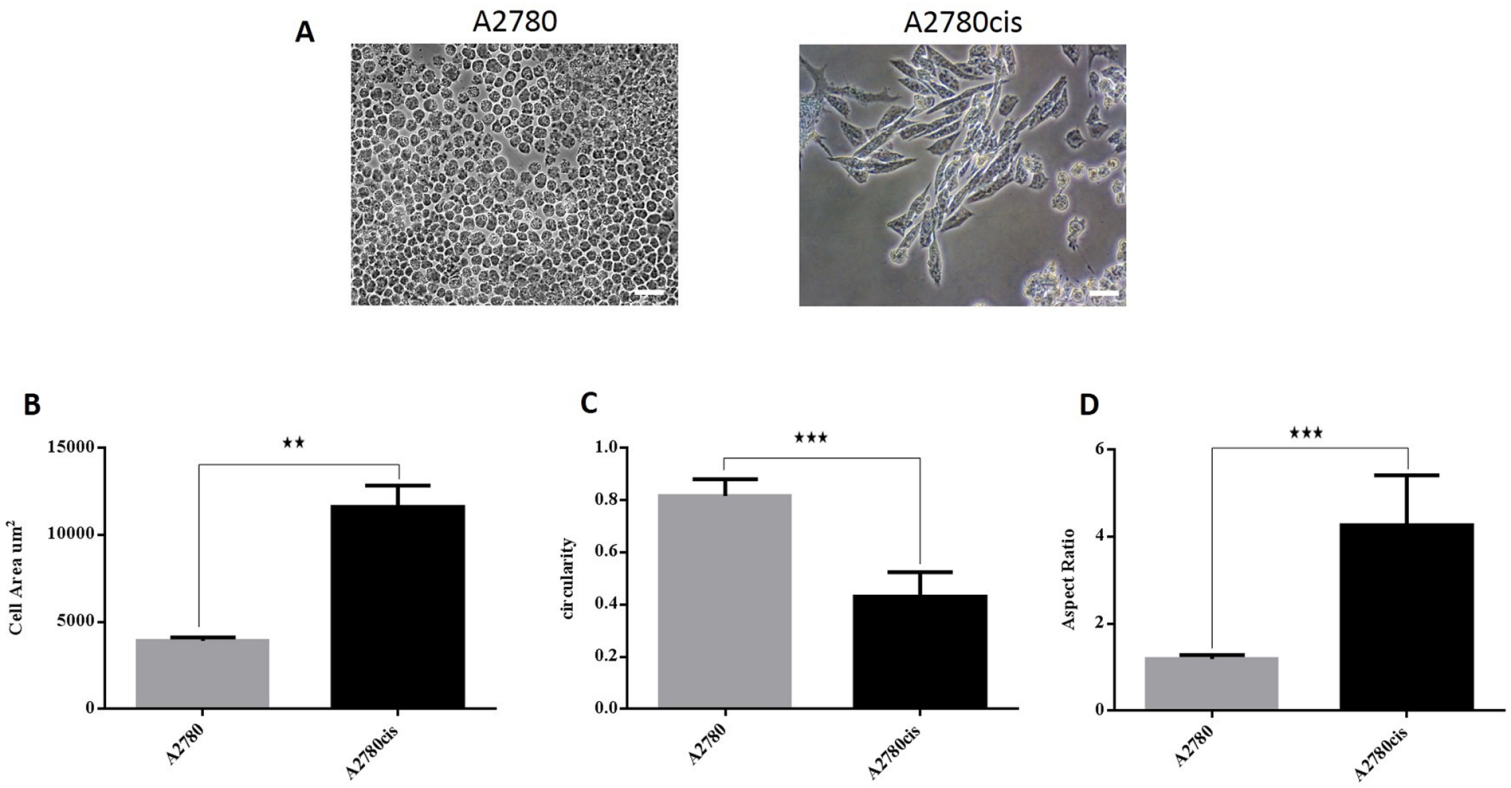
Cellular shapes of A2780cis and A2780 cells. (**A**) A2780 cells showed small round-shaped cells and A2780cis exhibit larger, more elongated shaped cells. Scale bar = 200 µm. (**B**) The mean cell area of A2780 and A2780cis cells shows that A2780cis cells have a larger area compared to A2780. (**C**) Cell circularity confirms that A2780 cells have a higher (mean) circularity value compared to A2780cis. (**D**) A2780cis have an aspect ratio above 4 while the round shape of A2780 is reflected by an aspect ratio ~ 1.

Figure 1 shows the morphological characteristics of the different cancer cell lines’ sensitivity to chemotherapy during exponential growth, i.e., the shape and size of the cells during culture. A2780 cells showed a small round shape with a cell area of 3915 μm^2^, a circularity of 0.81 and an aspect ratio of 1.19. However, A2780cis cells were larger and more elongated with a cell area of 11,600 μm^2^ with a circularity of 0.43 and an aspect ratio of 4.26 (Fig. 1A–D).

**Figure 2:**
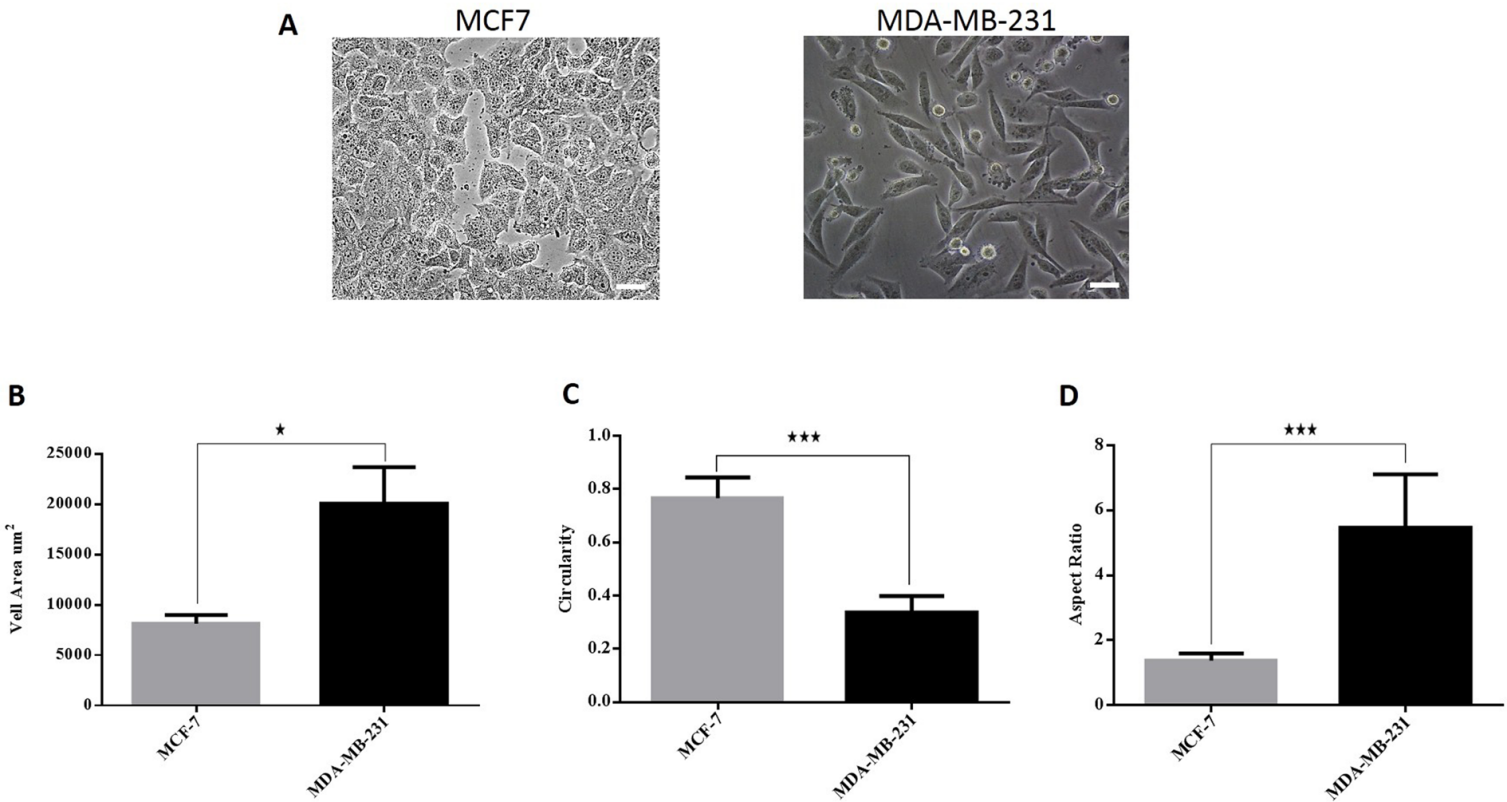
Cellular shapes of MDA-MB-231 and MCF7 cells. (**A**) MCF7 cells showed squamous epithelial cell shape and MDA-MB-231 exhibit spindle-like morphology. Scale bar = 200 µm. (**B**) The mean cell area of MCF7 and MDA-MB-231 cells shows that MDA-MB-231 cells have a larger area compared to MCF7. (**C**) Cell circularity confirms that MCF7 cells have a higher (mean) circularity value compared to MDA-MB-231. (**D**) MDA-MB-231 cells have an aspect ratio above 5 while the round shape of MCF7 is reflected by an aspect ratio ~ 1.

MDA-MB-231 and T98G cell lines were used as reference cells since they are well-known to be resistant to chemotherapy^45–47^. Figure 2A shows that MCF7 cells have a small squamous appearance with an 8128 μm^2^ cell area, a circularity of 0.77, and an aspect ratio of 1.37. Characterized by larger and more elongated and spindle-like morphology, MDA-MB-231 cells showed a cell area of 20,050 μm^2^ with a mean circularity of 0.33 and a mean aspect ratio of 5.47 (Fig. 2B–D).

**Figure 3:**
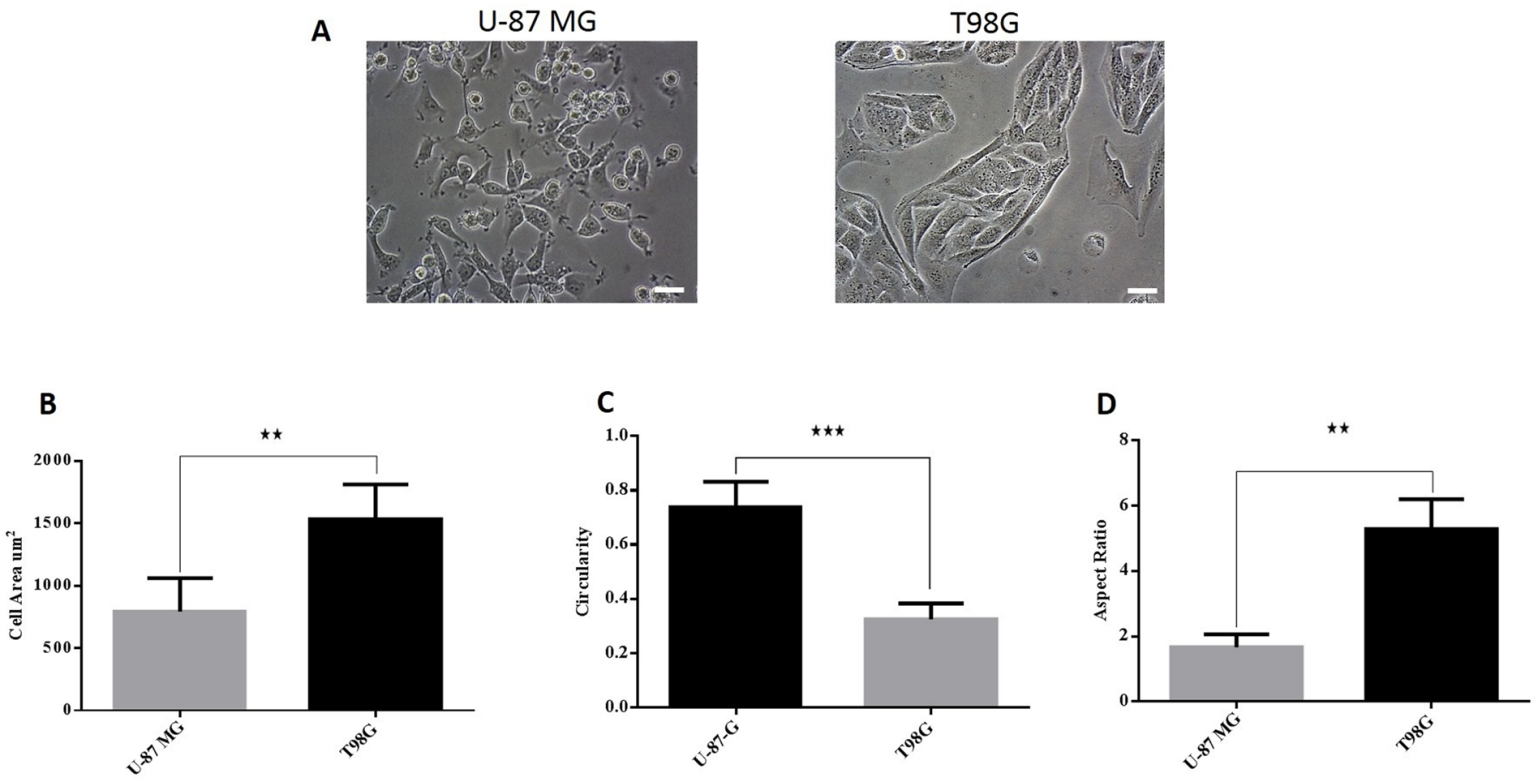
Cellular shapes of T98G and U-87 MG cells. (**A**) U-87 MG cells and T98G cells showed spindle-like morphology. Scale bar = 200 µm. (**B**) The mean cell area of U-87 MG and T98G cells shows that T98G cells have a larger area compared to U-87 MG cells. (**C**) Cell circularity confirms that U-87 MG cells have a higher (mean) circularity value compared to T98G cells. (**D**) T98G cells have a higher aspect ratio ~ 5 than U-87 MG (aspect ratio ~ 2).

Both U-87 MG and T98G cells exhibit spindle-like morphology, where T98G cells showed a larger and more elongated cell shape than U-87 MG (Fig. 3A). U-87 MG and T98G cells demonstrated cell areas of 793 and 1535 μm^2^, with a circularity of 0.73 and 0.32 and an aspect ratio of 1.67 and 5.28, respectively (Fig. 3B–D).

### Dissimilar shaped cancer cell lines have different cisplatin tolerance capacity

**Figure 4:**
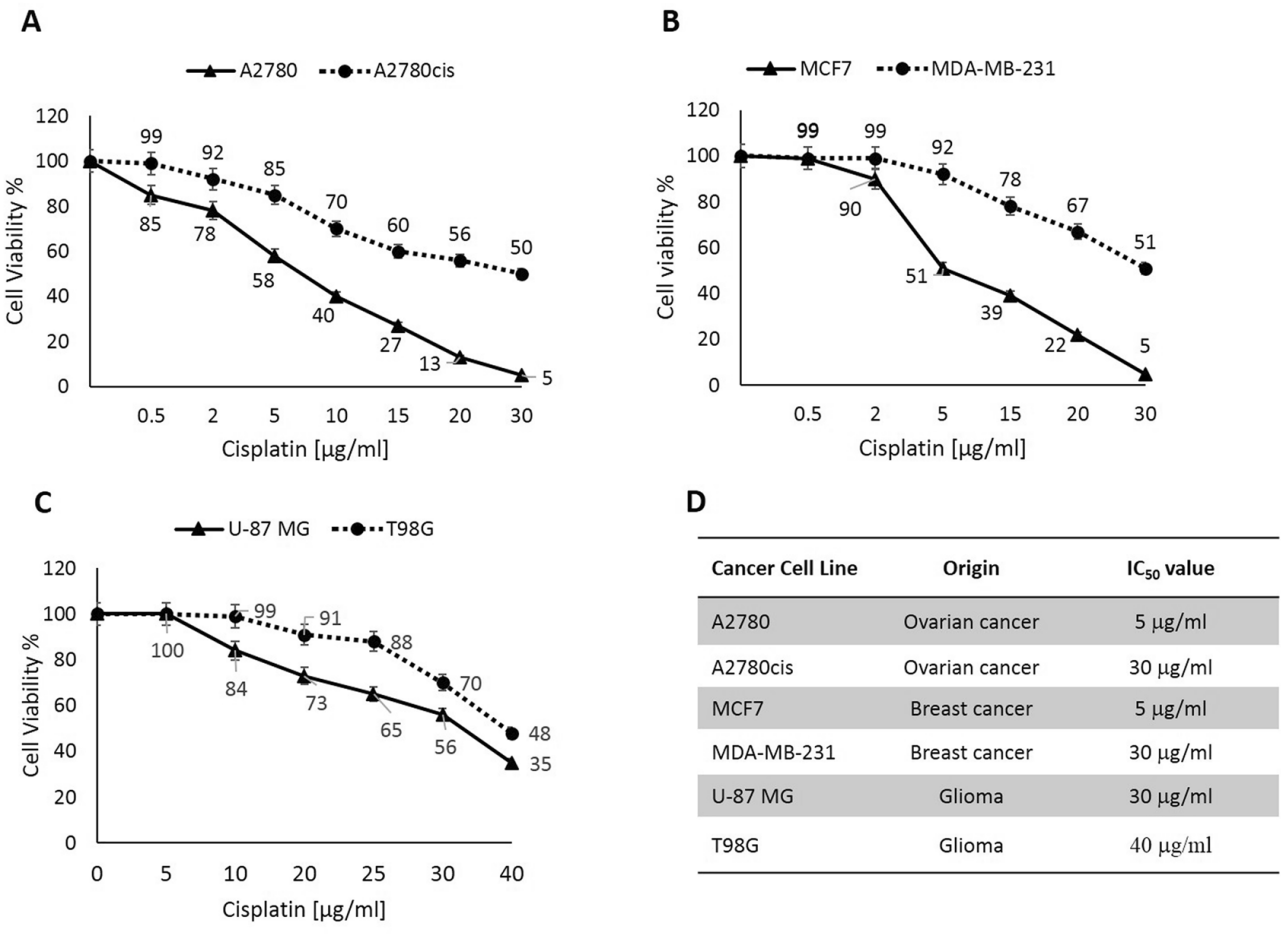
Cancer cells exhibit different resistance to cisplatin-induced cytotoxicity. (**A**): A2780 and A2780cis cells. (**B**): MCF7 and MDA-MB-231 cells. (**C**) U-87 MG and T98G cells. (**D**) Table showed IC_50_s of the studied cell lines.

MTT assay was performed to assess the effect of cisplatin on different human cancer cell lines. Cells were treated with increased concentrations of cisplatin for 24 h. The concentration-dependent effect of cisplatin on various cells was observed. IC_50_ value in A2780cis cells was four times higher than the parental cell line’s IC_50_, suggesting that the A2780cis cells are more resistant to cisplatin-induced cytotoxicity compared to A2780 cells (Fig. 4A). Results in Fig. 4B showed that the IC_50_ value for MDA-MB-231 cells is six times greater than IC_50_ for MCF7 cells. Figure 4C shows the differences in cisplatin sensitivity between U-87 MG and T98G cell lines. IC_50_ in T98G cells was 1.3 times higher than U-87 MG’s IC_50_. The IC_50_ results were summarized in Fig. 4D.

### MDR1 expression increases in the resistant variant A2780cis cell line

ABC transporter genes are responsible for chemotherapy cellular response. Therefore, assessing the mRNA expression level of *MDR1 (multidrug resistance protein 1 gene)*, *MRP1* (MDR-related protein 2) and *MRP2* (MDR-related protein 2) using qRT-PCR might help understanding if there is a correlation between cisplatin tolerance and expression of ABC transporter genes in A2780cis cell line.

**Figure 5:**
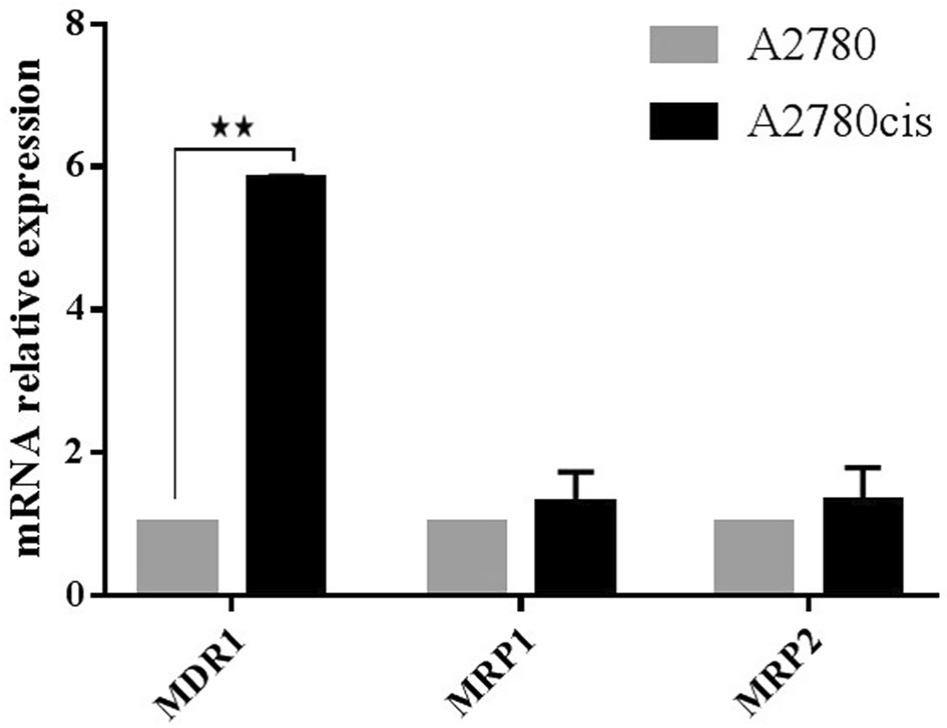
qPCR analysis of the *MDR* genes in A2780cis cells. *MDR1* Expression increases in the resistant variant A2780cis cells. The expression levels were normalized to parental cells A2780.

Figure 5 shows a statistically significant increase of *MDR1* transcript level (5.8 fold) in A2780cis cell line compared to the parental cell line. On the other hand, *MRP1* and *MRP2* expression did not significantly change in A2780 and A2780cis cell lines.

### Cisplatin resistant cancer cells have molecular changes consistent with EMT

**Figure 6:**
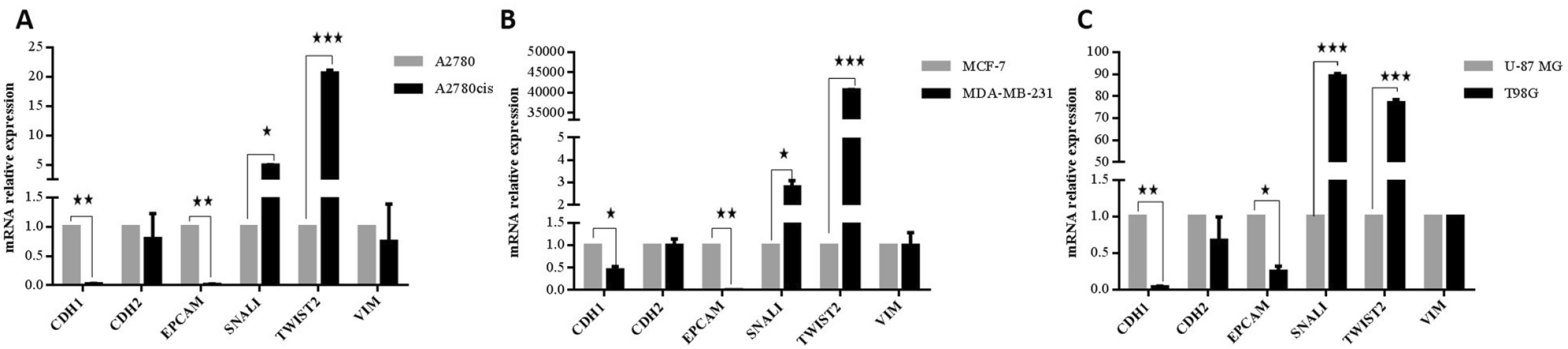
Resistant cancer cells display molecular changes consistent with partial EMT. Down-regulation in epithelial genes: *CDH1* and *EPCAM*, and up-regulation in mesenchymal marker *SNAIL* and *TWIST2*, were assessed using qPCR, no significant expression changes in *CDH2* and *VIM* genes were observed. (**A**) A2780cis compared to parental cells A2780. (**B**) MDA-MB-231 cisplatin resistant cells compared to MCF7 cisplatin sensitive cells. (**C**) T98G cisplatin resistant cells compared to U-87 MG the more sensitive cisplatin cells.

To determine whether the gaining of cisplatin resistance promotes specific molecular alterations corresponding with EMT in ovarian cancer, qRT-PCR was performed to investigate EMT-related biomarkers expression. Results showed that the expression of epithelial markers, *CDH1,* and *EPCAM*, was significantly reduced by 0.02 and 0.013 fold, respectively in the A2780cis cells compared with A2780 cells. The expression of mesenchymal markers, *SNAIL*, and *TWIST2*, were higher by 4.9 and 20.3 fold, respectively in the A2780cis cells compared with the parental cell line. Still, no significant expression changes in *CDH2* nor *VIM* genes could be observed (Fig. 6A). Additionally, no significant expression changes in other markers of EMT such as *ZEB1*, *ZEB2*, *SLUG*, *TWIST1* (S1) were observed. Based on these observations, A2780cis cells may be considered to have a hybrid mesenchymal epithelial phenotype.

The changes in transcriptional levels in MDA-MB-231 and T98G cells were compared to find out if the changes observed in A2780cis are applied in other cancer cells resistant to therapy. In a similar pattern to A2780cis cells, The EMT markers *CDH1* and *EPCAM* genes were reduced by 0.5 and 0.006 fold, respectively, in MDA-MB-231compared to MCF7 cells. These reductions were associated with up-regulation of EMT genes *SNAIL* and *TWIST2* (2.6 and 40,732 fold). However, no significant changes were observed in *CDH2* nor in *VIM* expression in MDA-MB-231 in comparison with MCF7 cells (Fig. 6B) nor other markers of EMT (S1). The same molecular changes were observed in EMT markers; *CDH1* and *EPCAM* were down-regulated (0.046 and 0.2 fold, respectively) in T98G, the more resistant cell line, whereas *SNAIL* and *TWIST2* (88.5, 76 folds, respectively) were up-regulated (Fig. 4C). No significant changes were observed in CDH2 nor VIM expression (Fig. 6C) nor other markers of EMT (S1) in T98G in comparison with U-87 MG cells. A significant overlap was noted in expression profiles of EMT genes between resistant cancer cell lines, proposing a shared mechanism associated with resistance to therapy.

### 5-azacytidne treatment up-regulates expression of EMT-related genes in A2780 cell line

**Figure 7:**
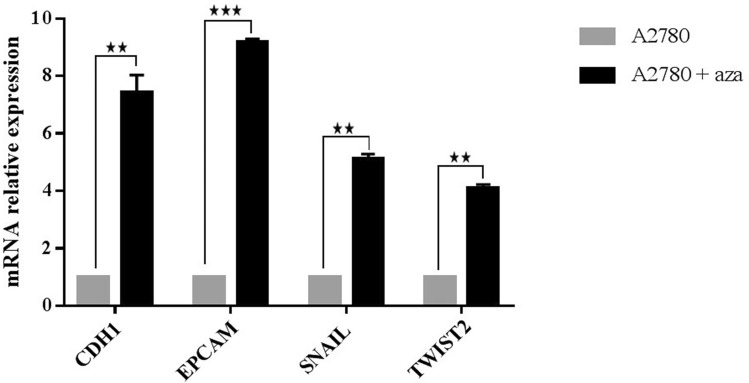
EMT marker gene expression changes after exposure to the demethylating agent 5-azacytidine. qPCR validation of gene expression showed a significant up-regulation of EMT genes in A2780 cells treated with azacytidine compared with control.

Azacytidine treatment was applied to identify the epigenetically regulated genes from the gene set that expressed differentially between A2780 cell line and its resistant variant. The gene expression analysis by qPCR showed that azacytidine treatment significantly increased the expression of *CDH1*, *EPCAM*, *SNAIL*, and *TWIST2* genes, by 7.8, 9.26, 5.24, and fourfold, respectively (Fig. 7). This suggests that DNA methylation may be essential in the regulation of the expression of these genes in this cell line.

### Acquisition of cisplatin resistance in A2780 cell line induced aberrant DNA methylation in EMT-related genes

Methylscreen assay was used to determine the DNA methylation profile in genes that are differentially expressed between A2780 and resistant variant cells and that expression increased after azacytidine treatment. PCR primers were designed to amplify genomic DNA at the TSS associated CpG islands of these genes. The PCR amplicons ranged from 151 to 523 bp in length. These amplicons contained different site numbers for HhaI/HpaII, AciI and McrBC enzymes. The size of DNA fraction amenable to digestion (analytical window) determines assay sensitivity and it was represented by ΔCt between the Rsd and R0 reactions and it ranged from 3.2 to 13.6.

**Figure 8:**
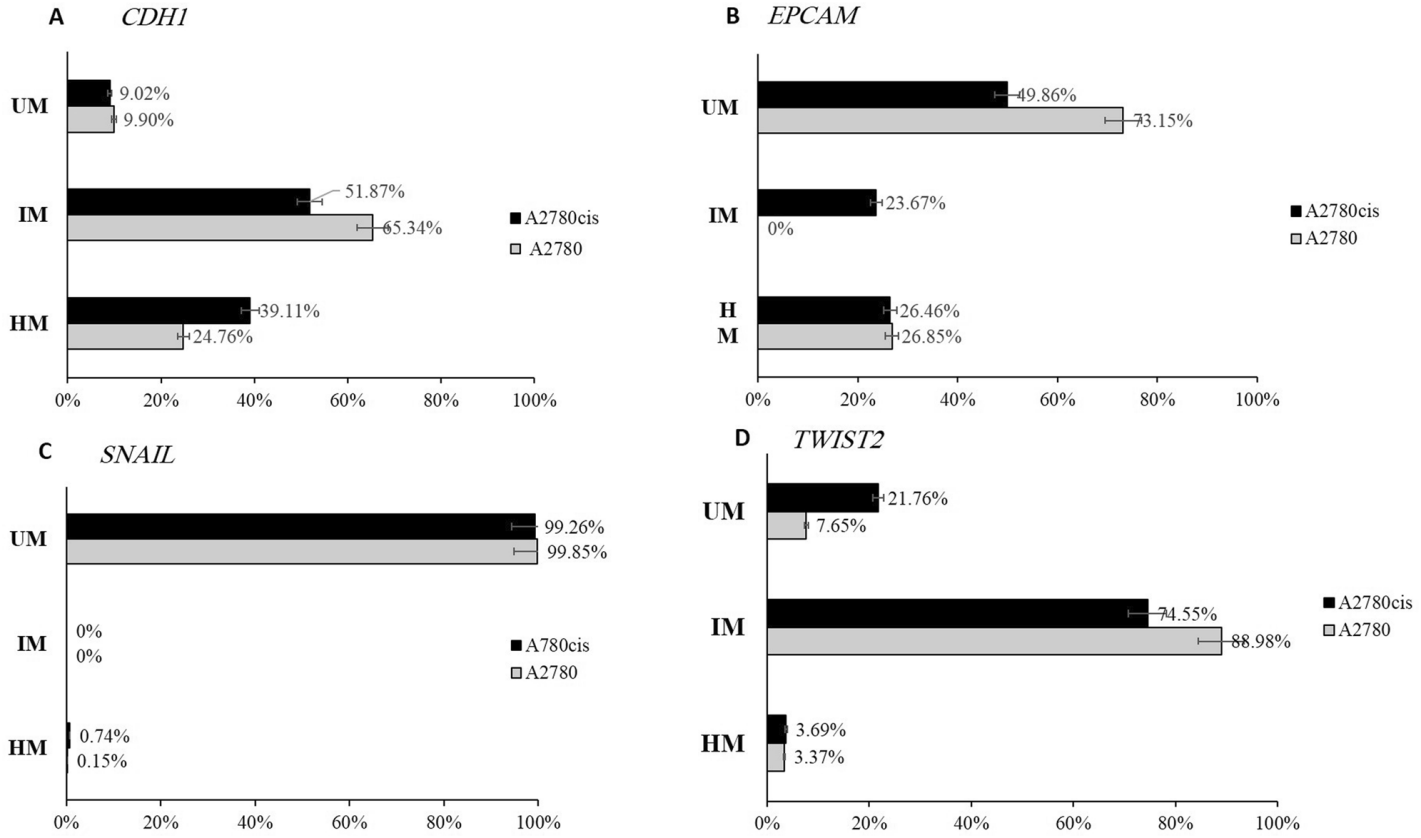
Methyscreen assay for *CDH1*, *EPCAM*, *SNAIL*, and *TWIST2* in A2780 and A2780cis cell lines. (**A**) Charts display the result of *CDH1* assay as a percentage of each portion of DNA. (**B**) Charts display the result of *EPCAM* assay as a percentage of each portion of DNA. (**C**) Charts display the result of *SNAIL* assay as a percentage of each portion of DNA. (**D**) Charts display the result of *TWIST2* assay as a percentage of each portion of DNA.

The charts display the result of each assay as a percentage of each portion of DNA according to its methylation state, i.e., the unmethylated fraction, intermediate methylated and hypermethylated fraction (Fig. 8). Results from *CDH1* assay revealed a 13.47% hypermethylation in the densely methylated portion after the acquisition of cisplatin resistance. The methylation of the region from (− 306 to − 82 bp), which contains [7 Acil, 3 HpaII and 7 MCrBc] restriction sites, increased from 24.76 to 39.11% in A2780 and A2780cis, respectively (Fig. 8A). On the other hand, Fig. 8B shows that the CpGs in the region (− 463 to − 296 bp) of *EPCAM* gene that contains [8 AciI, 4 HhaI, and 7 MCrBc] restriction sites in A2780 DNA were 73.15% unmethylated, 0% intermediate methylated and 26.85% densely methylated. In A2780cis the unmethylated portion value had decreased and CpGs had gained methylation by 23.67%. Methylscreen assay of *SNAIL* gene revealed that (− 688 to − 165 bp) region is unmethylated in A2780 cell line and no significant differences in methylation between A2780cis and its parental cell line (Fig. 8C). The methylation analysis of *TWIST2* gene revealed that the region from (− 328 to − 177 bp) that contains [6 AciI, 3 HhaI, and 4 MCrBc] restriction sites was 3.37% hypermethylated, 88.98% intermediate methylated and 7.65% unmethylated. In A2780cis, In A2780cis, the fraction of unmethylated DNA was 21.76% (Fig. 8D) and the intermediate methylated portion value had diminished compared to A2780.

### 5-azacytidne and cisplatin treatment induces cell shape changes and partial reverse EMT

**Figure 9:**
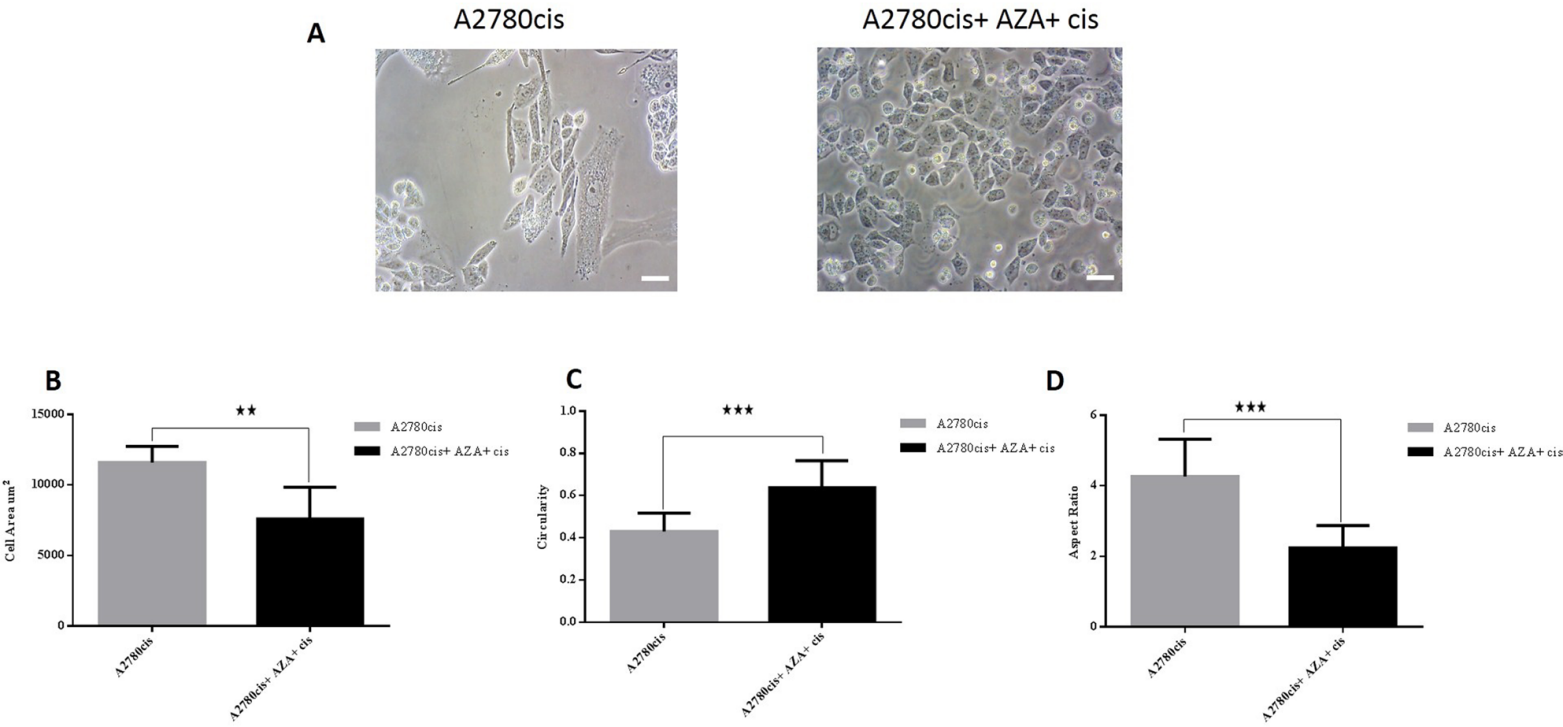
A2780cis cancer cell line treated with aza and cisplatin displays morphological changes associated with partial MET. (**A**) A2780cis cells showed elongated shaped cells and treated cells exhibit smaller, more circular shaped cells. Scale bar = 200 µm. (**B**) The mean cell area of A2780cis and A2780cis treated cells shows that treatment cause a reduction in cell size. (**C**) Cell circularity confirms that A2780cis treated cells have a higher (mean) circularity value compared to A2780cis. (**D**) Treatment of A2780cis with aza and cisplatin reduces the aspect ratio to 2.

**Figure 10:**
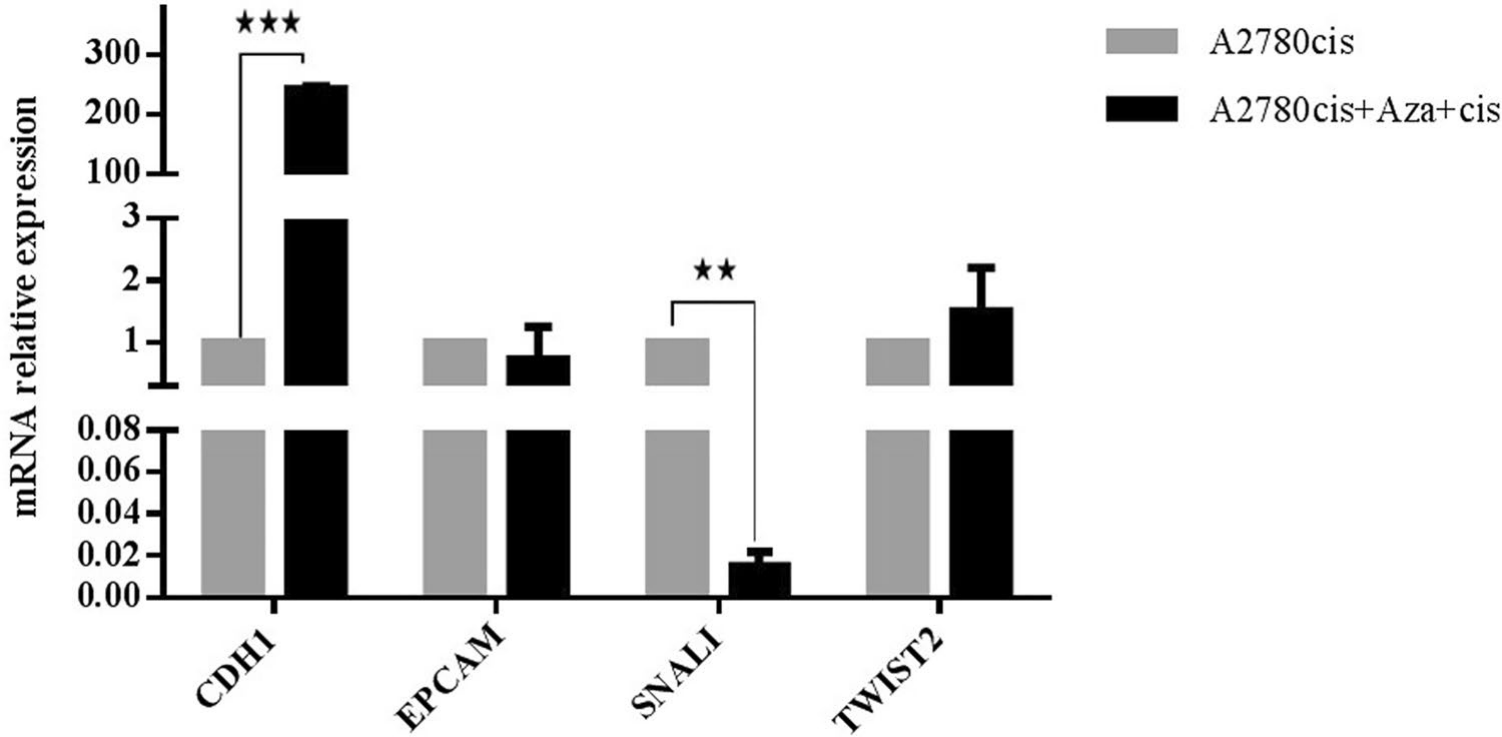
Treatment of A2780cis cells with aza and cisplatin causes molecular changes consistent with partial MET. Up-regulation in epithelial gene *CDH1*, and down-regulation in mesenchymal marker *SNAIL*, were assessed using qPCR. No significant changes in *EPCAM* and *TWIST2* gene expression were observed in A2780cis treated cells.

To test whether DNA methylation changes are causally underlying EMT induced by acquired chemoresistance. In this respect, A2780cis cells were exposed to noncytotoxic doses of the demethylating agent 5-aza, followed by cisplatin. The morphological characteristics of A2780cis were compared before and after treatment. 5-azacytidne and cisplatin treatment reduces cell area by 40%, increases circularity from 0.43 to 0.64 and decreases aspect ratio from 4.26 to 2.24 in treated cells compared to control cells (Fig. 9). These results indicate that A2780cis cells undergo shape changes towards epithelial shape after aza and cisplatin treatment. The expression of epithelial marker *CDH1* was significantly upregulated by 246 fold in treated cells compared with the control group. The expression of mesenchymal markers, *SNAIL* was significantly diminished in the A2780cis cells compared with the parental cell line. No significant expression changes in the epithelial marker *EPCAM* nor the mesenchymal markers *TWIST2* were observed in treated A2780cis cells compared to control cells (Fig. 10). These data indicate that the treatment of demethylating agent 5-azacytidne followed by cisplatin cause a partial reverse of EMT with cell shape changes.

## Discussion

Ovarian cancer ranks as one of the most common causes of cancer deaths among females^1^. Patients suffering from ovarian cancer have a poor prognosis, with a low survival rate^4^. High mortality of ovarian cancer is mainly due to metastasis and the evolution of resistance to chemotherapies^3^. Cisplatin is a cornerstone of the treatment regime for many solid tumors, including ovarian cancer^5,6^. However, its clinical effectiveness is influenced by tumor cells acquiring chemoresistance^9^. Many studies concentrated on the molecular mechanisms mediating the development of cisplatin resistance have identified decreased cellular uptake of the drug, increased drug efflux, enhanced DNA damage repair capacity, and anti-apoptotic signaling as relevant pathways^10,11^.

In this study, it was demonstrated that the A2780cis cell line still has the tolerance capacity to cisplatin and its resistance is accompanied by an increase in the expression of *MDR1*. This marker seems to be a universal cellular response marker to chemotherapy in various cancers. A high expression level of *MDR1* was detected in the cisplatin, paclitaxel and doxorubicin resistant variant of A2780 cells and different resistant cell lines derived from ovarian cancer^48–51^. Several studies have shown conflicting results about the differential expression of *MRP1* and *MRP2* genes between sensitive and resistant ovarian cancer cell lines, and the ability of these changes to represent the chemoresistance^50,52^. Recently, epithelial-mesenchymal transition (EMT) was implicated as a core mechanism mediating drug resistance^53^. EMT is a biological mechanism characterized by loss of cell adhesion, as well as loss of cell polarity and gain motility^18^. EMT results in changes in cell morphology associated with alterations in epithelial and mesenchymal markers expression^54^. EMT like phenotype has been reported in chemoresistant variant cell lines generated upon multiple rounds of chemotherapy treatment such as; cisplatin resistant ovarian cancer cells (TOV-112D, MDAH, OVSAHO, SKOV-3/DDP, OVCAR3/DDP and A2780CP)^12–15^, A2780/PTX, NOS-PR, TAOV-PR, and SKOV-PR paclitaxel resistant ovarian cancer cells^16,17^. Similarly, chemotherapeutic resistance promotes EMT like changes in other cancer cell lines, including non-small cell lung cancer^28,29^, gastric cancer^27^ nasopharyngeal carcinoma cells^30^, and breast cancer cells^31,32^. Partial EMT has been proposed as an important mechanism in cancer metastasis and chemoresistance^55–57^. Cells that have partial EMT phenotype display properties of both epithelial and mesenchymal cells, indicating that EMT is being induced but not completed^58^. Galle et al. discussed the role of partial EMT in resistance acquisition, they showed that 3 ovarian cell lines (U10, U100, and IGROV-1/CDDP) underwent partial EMT when they acquired chemoresistance^42^. In this present study, it was proven that the resistant variant A2780cis cell line underwent partial EMT. This was confirmed by observing a morphological change from small circular shaped to elongated shaped cells and changes in molecular markers of EMT; significant reduction in *CDH1* and *EPCAM* and upregulation of the transcription factors, *SNAIL* and *TWIST2*. Additionally, no significant expression changes were observed in the studied mesenchymal markers such *CDH2, VIM*, ZEB*1*, *ZEB2*, *SLUG*, *TWIST1* that should be upregulated in completed EMT.

Multiple studies have shown that naïve cancer cell lines and clinical tumor samples can be divided according to their mesenchymal/epithelial phenotype, this sorting could determine the sensitivity to chemotherapy in various cancers, including ovarian, breast, and lung cancers^59–64^. From this observation, The EMT like changes detected in A2780cis were compared with changes in breast and glioma cell line models known as resistant cancer cells^45–47^. MDA-MB-231 and T98G cancer cells were used as reference cell lines models to figure if the acquired changes observed in A2780cis are common in other cancer cells, resistant to therapy. The same molecular changes consistent with morphological alteration in cisplatin resistant MDA-MB-231 cells were detected compared with the sensitive MCF7 cells. The same transcripts changes in *CDH1*, *EPCAM*, *SNAIL* and *TWIST2* genes were also detected in chemoresistant T98G cell line in comparison with the less cisplatin tolerance cells U-87-MG. This indicates the importance of *CDH1*, *EPCAM*, *SNAIL* and *TWIST2* genes in EMT mechanism associated with cisplatin resistance. Similarly, no significant expression changes were observed in the studied mesenchymal markers in the resistant cancer cells such *CDH2, VIM*, ZEB*1*, *ZEB2*, *SLUG*, *TWIST1* that which are upregulated in completed EMT. These data indicate that these cell lines have a partial EMT state.

*CDH1* is a Ca^2+^ dependent adhesion molecule that binds by its extracellular domain to *CDH1* on the adjacent cell creating a bridge between the cell’s cytoskeletons^65^. Many studies revealed that *CDH1* down-regulation may be associated with cancer cells resistant to chemotherapy that can be attributed to the EMT mechanism activation^35,66^. Acquisition of paclitaxel chemoresistant induces EMT phenotypic changes and *CDH1* down-regulation in NOS-PR and A2780/PTX ovarian cancer cell line^16,17^. EPCAM is an epithelial cell surface transmembrane glycoprotein that mediates homophilic cell–cell adhesion^67,68^. In ovarian cancer cells, *EPCAM* upregulation is connected to a more favorable prognosis and more effective platinum-based therapy^69^. Galle et al., found *EPCAM* expression is down-regulated in addition to *CDH1* in resistant variant cancer cells due to EMT process activation^42^. EMT transcription factors such as *SNAIL* are considered as direct repressors of *CDH1* as they bind to E-boxes existing on the *CDH1* promoter^70–72^. Hojo et al., observed that ovarian cancer cell lines OVCAR8 and COV318 with high Snail/CDH1 showed more motile and cisplatin resistant phenotypes than OVSAHO and Kuramochi cell lines that have low *SNAIL*/*CDH1*^59^. TWIST2 is considered as a direct repressor of *CDH1*, it can bind directly on E-boxes existing on the *CDH1* promoter to suppress its expression and it can repress *CDH1* expression indirectly through activation of other signaling pathways^73^. Studies show that *TWIST2* expression is a prognostic indicator for overall survival and disease-free survival and its overexpression correlates with poor prognosis and is associated with *CDH1* down-regulation giving mesenchymal cell phenotype on ovarian cancer tumors^74,75^. Wang et al., demonstrate that *TWIST2* plays a critical role in the cisplatin resistance of ovarian cancer. They found that *TWIST2* expression was up-regulated in resistant variant C13K ovarian cancer cell line compared to the cisplatin sensitive ovarian cancer cell line OV2008^76^.

DNA methylation is one of the best described epigenetic mechanisms that regulate gene expression. Aberrant DNA methylation is observed in CpG dinucleotides clustered around the TSS of genes, called CpG islands, leading to gene expression dysregulation^77^. Upon initiation of EMT, DNA methylation of the genome selectively undergoes CpG site methylation changes, which regulate transcription of EMT-related genes^35,42,78,79^. In the current study, the investigation of methylated CpG islands role in the modulation of gene expression of EMT regulated genes was done in A2780 cancer cell lines. DNA methyltransferase inhibitor 5-aza induces gene expression of *CDH1*, *EPCAM*, *SNAIL*, and *TWIST2,* which indicates that DNA methylation may regulate these genes. Chang et al., used gene expression profiling after cancer cells treatment with 5-azadeoxycytidine, they identified genes that were dysregulated in cisplatin resistant cancer cells and reactivated by the DNA methyltransferase inhibitor^80^. Here, it found that epithelial gene promoters *CDH1* and *EPCAM* became significantly more methylated in A2780cis compared to the parental cell line. These promoter methylation changes correlate with significant gene expression down-regulation. Boettcher et al., profiled DNA methylation of 800 selected CpG islands and identified hypermethylation in *CDH1* CpG islands in breast and ovarian doxorubicin resistance cancer cells^35^. *EPCAM* overexpression has been linked to promoter hypomethylation *EPCAM*-negative cells treated with a DNA methyltransferase inhibitor prompted *EPCAM* expression in various cancers types including ovarian cancer^81–83^. A recent study reported consistent methylation changes across multiple cancer cell lines that differed in chemoresistace. Specifically, hypermethylation of epithelial marker genes such as *CDH1* and *EPCAM* promoters and hypomethylation of mesenchymal marker genes such as *SNAIL* in resistant versus parental cell lines^42^. Analysis of *SNAIL* promoter region predicted a CpG island surrounding the TSS, the DNA methylation status in the genomic region (− 688 to − 165 bp) was examined, no methylation was observed in CpGs located in this region of *SNAIL* promoter in A2780 and its resistant variant cells, although there were changes in *SNIAL* expression between the two cell lines and after azacytidine treatment. Literature has described changes in the histone modifications regulating *SNAIL* gene expression^84^. A single study described changes in the methylation of CpG island located in the first intron after 1000 pb from TSS in EMT models of cancer cells^85^. The differential methylation in *SNAIL* gene could be possibly identified in the intron region in A2780 cell line. CpGs island of mesenchymal transcription factor *TWIST2* promoter showed DNA hypomethylation in A2780cis compared to the parental cells, this hypomethylation coincides with gene expression up regulation due to EMT activation. *TWIST2* methylation changes were observed in various cancers such as colorectal cancers, prostate cancer, and chronic lymphocytic leukemia, this epigenetic event might be the underlying mechanism for *TWIST2* transcriptional regulating^86,87^.

To test whether DNA methylation changes are causally underlying EMT induced by acquired chemoresistance, A2780cis cells were exposed to noncytotoxic doses of the demethylating agent 5-aza under the pressure of cisplatin. The changes in cell shape of A2780cis cells towards epithelial shape was observed. This treatment caused a reversal in the expression pattern of two EMT markers (*CDH1*, *SNAIL*) which indicates that the treatment of demethylating agent under the pressure of cisplatin cause a partial reversing of EMT with cell shape changes. This causality of methylation changes, EMT and cell shape changes caused by acquired chemoresistance to cancer therapy were noted in Galle et al. study by exposing treatment-resistant cell lines during cell divisions to the demethylating agent 5-aza-2′-deoxycytidine, and observing the reversing of EMT^42^.

There are several limitations in our study, like studying the direct effect of DNMTs inhibition, the methylation changes of the EMT gene and shape changes on the cellular cisplatin resistance. Similarly, the study of reversing EMT phenotype by suppressing the expression of EMT genes and its effect on cisplatin sensitivity in the resistant cell lines.

In conclusion, this study has shown, the gain of cisplatin resistance in cancer cells is accompanied by EMT-like changes at the morphological and molecular levels. It showed that DNA methylation changes of *CDH1*, *EPCAM* and *TWIST2* genes underlie the EMT induction in cisplatin resistant ovarian cancer cell line. Further evaluation is needed in future clinical studies to determine potential EMT associated epigenetic biomarkers for resistant phenotypes.

## Methods

### Cell culture

Human ovarian cancer cell lines A2780 (cisplatin sensitive human epithelial ovarian cancer cell line) and A2780cis (the resistant variant), human breast cancer cell lines MCF7 and MDA-MB-231, and human glioblastoma cancer cell lines U-87 MG and T98G were purchased from European Collection of Authenticated Cell Cultures (England). A2780 and A2780cis cell lines were grown in complete RPMI-1640 medium containing 10% FBS, 2 mM glutamine, 0.1 mg/ml each of penicillin and streptomycin. To maintain A2780cis resistance to cisplatin, 1 μM of cisplatin (Sigma-Aldrich, St. Louis, USA) was added to the media every 3 passages. MCF7 and MDA-MB-231 cell lines were grown in EMEM containing 10% FBS, 2 mM glutamine, 1% non-essential amino acids (NEAA), 0.1 mg/ml each of penicillin and streptomycin. U-87 MG and T98G cell lines were cultured in EMEM, 2 mM Glutamine, 10% FBS and 1% Sodium Pyruvate (NaP). All cell cultures were kept in a 5% (v/v) CO_2_ humidified atmosphere at 37 °C (Binder, Germany). Morphological phenotypes of cell lines were assessed when the cell density was up to 70% confluence using Eclipse TS100 inverted light microscope (Nikon, Japan). The Shape descriptors of the cells were assessed using Fiji image analysis software^88^, three parameters were investigated: cell area for cell size, circularity which is defined as 4*(area/perimeter^2^) and aspect ratio which is defined as the ratio of the major to the minor axis of a fitted ellipse as a measure of cell shape. Particles that have perfect circular shapes have circularity and aspect ratios equal to 1. Values of aspect ratios that are higher than 1 are associated with elongation and lower circularity values are associated with cellular bumps.

### Cell viability assay

Cells were plated into 96-well-plates (1 × 10^4^ cells/well) for MTT assay and allowed to attach O/N. Different concentrations of cisplatin were used to treat cells for 24 h. MTT solution (Roche, Germany) was added to each well and incubated for 4 h. at 37 °C. Then absorbance values were measured at 550 nm using Multiskan Ascent absorbance plate reader (Thermo Labsystems, Germany). Cell viability was determined as follows:

$$ {\text{Cell}}\;{\text{viability}}\left( \% \right) = \left( {{\text{average}}\;{\text{OD}}\;{\text{value}}\;{\text{of}}\;{\text{experimental}}\;{\text{group}}/{\text{average}}\;{\text{OD}}\;{\text{value}}\;{\text{of}}\;{\text{control}}\;{\text{group}}} \right)*{1}00\% . $$

### Gene expression analysis by qRT-PCR

Total RNA was extracted from A2780, A2780cis, MCF7, MDA-MB-231, U-87 MG, T98G cell lines, from A2780 treated with 5-azacytidine (5-aza), and from A2780cis treated with cisplatin and 5-aza using the RNeasy Mini kit (Qiagen, Germany). First-strand cDNA synthesis was carried out from 3 μg total RNAs were reverse transcribed to cDNAs using M-MLV reverse transcriptase (Invitrogen, USA) for 2 h. at 37 °C. To calculate the relative expression of EMT regulating genes; (*CDH1*, *CDH2, EPCAM*, *SNAIL1*, *TWIST2, VIM*), and ABC transporters (ATP-binding cassette transporters) genes; (*MDR1-* multidrug resistance protein 1 gene, *MRP1-* MDR1-related protein 1, *MRP2-* MDR-related protein 2), quantitative real-time PCR was performed using Maxima™ SYBR™ Green/ROX 2 × qPCR Master Mix (Thermo Scientific, USA) for 40 amplification cycles using StepOne™ Real-Time PCR System (Applied Biosystems, USA). Relative transcript fold changes were calculated using the ΔΔCt method with *GAPDH* as a reference gene. All reactions were run in triplicate. Primers sequences are detailed in Tables 1 and S2.

**Table 1:** Primer sequences used for quantitative real-time PCR in this study.

Gene symbol	Primer’s sequence (5′–3′)
*MDR1*	F-GAGGGGATGGTCAGTGTTGATGG
	R-ATCGTGGTGGCAAACAATACAGGT
*MRP1*	F-CTCCTGTGGCTGAATCTGGGC
	R-AGCACTTTGATCCCATTGAGAATTTCG
*MRP2*	F-CCTGGGAACATGATTCGGAAGCC
	R-GGAGGATTTCCCAGAGCCGAC
*CDH1*	F-GTGGGCCAGGAAATCACATCCTA
	R-GTTGGCAGTGTCTCTCCAAATCC
*CDH2*	F-TGGACGGTTCGCCATCCAGAC
	R-AGTCGATTGGTTTGACCACGGTG
*EPCAM*	F-GCCGCAGCTCAGGAAGAATGTG
	R-CAACTGAAGTACACTGGCATTGACG
*SNAIL*	F-TGCAGGACTCTAATCCAGAGTTTACC
	R-GGTGGGATGGCTGCCAGC
*TWIST2*	F-CAAGCTGAGCAAGATCCAGACGC
	R-GGTCATCTTATTGTCCATCTCGTCG
*VIM*	F-GCCGAAAACACCCTGCAATCTTTC
	R-CTCCTGGATTTCTCTTCGTGGAG
*GAPDH*	F-ATGACCCCTTCATTGACC
	R-GAAGATGGTGATGGGATTTC

### 5-Azacytidine treatment

In order to select the candidate genes for the methylation study, A2780 cells were cultured and treated with 0.1 μM 5-Azacytidine (Sigma-Aldrich, USA). The culture medium was removed every 24 h. and replaced with a fresh medium containing 0.1 μM 5-aza. Treated and mock treated cells were collected after 5 treatment days and total RNAs were extracted as described above.

### DNA extraction

Genomic DNAs from A2780 and A2780cis cells were extracted using the QIAamp DNA Mini kit (Qiagen, Germany) according to the manufacturer’s instructions. Isolated DNAs were quantified using NanoVue Plus (GE Healthcare Life Sciences, Germany).

### Methylscreen assay

Quantitative PCR-based methylation analysis (Methylscreen assay) was performed to analyze DNA methylation of genes that have differential expression between A2780 and the resistant variant cells and that expression increased after 5-aza treatment. Methylscreen assay is based on combined restriction digestion of DNA with methylation sensitive and methylation dependent restriction enzymes, MSRE and MDRE respectively^89^. Genomic DNA of A2780 and A2780cis cells were divided into four parts and treated with different digestions: (1) Rs: two methylation-sensitive enzymes MSRE (HhaI + AciI) or (HpaII + AciI) depending on the frequency of their restriction sites within the studied fragments, which are cutting only unmethylated DNA, (2) Rd: one methylation-dependent restriction enzyme McrBC (MDRE), which is cutting only methylated DNA or (3) Rsd: both MSRE and MDRE enzymes (double digest, DD), and (4) R0: neither MSRE nor MDRE (mock control). illustrations of the studied section of CDH1, EPCAM, SNAIL, and TWIST2 genes as shown in Supplementary (S3). Each 50 μl reaction contained 1 μg of gDNA, 1 × CutSmart Buffer, 100 μg/mL BSA, 1 mM guanosine-5′-triphosphate, 3% glycerol and 10 U of each enzyme used in restriction reaction, 50% glycerol was used instead of enzymes in mock reaction in order to keep restriction digest cocktail homogeneity. Digestions were incubated at 37 °C for 6 h. followed by inactivation of the enzymes at 65 °C for 20 min. The enzymes, CutSmart Buffer, BSA, and guanosine-5′-triphosphate were purchased from New England Biolabs, USA. Restricted samples were analyzed by qPCR with locus-specific PCR primers and SYBR Green dye. An *in-silico* analysis was performed using EMBOSS Cpgplot sequence analysis tool (https://www.ebi.ac.uk/Tools/seqstats/emboss_cpgplot/) from European Bioinformatics Institute (EMBL-EBI) to identify the CpG sites associated with the proximal promoter and transcription start site (TSS) for four genes. Sets of locus-specific PCR primers were designed to amplify gDNA at proximal CpG located within 1000 bp (±) of the transcription start site for each gene. Primers sequences, genomic loci, numbers of CpGs nucleotides and number of restriction sites contained in amplified amplicons are listed in Table 2. The PCR amplification was performed in 20 μl volume with 10 μl Maxima™ SYBR™ Green/ROX 2 × qPCR Master Mix (Thermo Scientific, USA), 300 nM of each primer and 2 μl (40 ng) of digested template DNA using the qPCR System. The PCR conditions were as follows: 95 °C for 10 min, and 45 cycles of 95 °C for 1 min and temperature for optimized annealing for 1 min. Amplification for each sample was performed in triplicate in a 48-well plate. All primer pairs were tested to identify the annealing temperature for optimal efficiency and melting curve analysis was conducted after the reaction to verify the amplification of the desired products.

**Table 2:** Primer sequences used for methylation study using methylscreen method.

Gene symbol	Number of CpGs and their locations	Product size (bp)	Number of enzymes restriction sites	Primer’s sequence (5′–3′)
*CDH1*	17 CpGs − 182 to 42	246	7 Acil 1 Hpa1 7 MCrBc	F-CAACTCCAGGCTAGAGGGTCAC
				R-ACTTCCGCAAGCTCACAGGTGC
*EPCAM*	18 CpGs − 107 to 82	189	8 Acil 4 Hha1 7 MCrBc	F-CTCCTCGGAGGCCACCAAAGAT
				R-CCGCTGGTGCTCGTTGATGAGT
*SNAIL*	65 CpGs − 688 to − 165	523	15 Acil 13 Hha1 19 MCrBc	F-AGAGGGCAGGGGTCTTCA
				R- AGATGAGCATTGGCAGCG
*TWIST2*	15 CpGs − 144 to + 7	151	6 Acil 3 Hha1 4 MCrBc	F-CCGAAGGGGGAGGCAAAACTGA
				R-ACTCTAGCTGGGCTGGGTTGCT

### Calculations of DNA methylation occupancy

The Ct values from R0, Rs, Rd and Rsd, reactions were used to calculate the initial amount of DNA in each digest before PCR as follows:

$$ {\text{CMs}} = {2}^{{ - {\text{Ct}}\left( {{\text{Rs}}} \right)}} ;\;{\text{CRd}} = {2}^{{ - {\text{Ct}}({\text{Rd}})}} ;\;{\text{CRsd}} = {2}^{{ - {\text{Ct}}({\text{Rsd}})}} ;\;{\text{CR}}0 = { 2}^{{ - {\text{Ct}}({\text{R}}0)}} . $$

The DNA methylation (%) was calculated as follows:

Hypermethylated DNA fraction (HM) = Rs/(R0 − Rsd) × 100; unmethylated DNA fraction (UM) = Rd/(R0 − Rsd) × 100; intermediately methylated DNA fraction (IM) = 1-HM-UM. If or ΔCt(Rd − R0) or ΔCt(Rs − R0) < 1.0, The DNA methylation (%) was calculate as following: HM = 1-UM, UM = 1-HM^89,90^.

### 5-Azacytidine and cisplatin combination treatment

In order to find out if DNA methylation changes are causally underlain EMT, A2780cis cells were cultured and treated with 0.2 μM 5-Azacytidine (Sigma-Aldrich, USA). The culture medium was removed every 24 h and replaced with a fresh medium containing 0.2 μM of 5-aza for 5 days, subsequently, cells were treated with 10 μg/ml cisplatin. Treated and mock treated cells were photographed and collected for RNA extraction and cDNA synthesis as described above. The relative expression changes of EMT regulating genes *CDH1*, *EPCAM*, *SNAIL1*, *TWIST2* were studied by qPCR.

### Statistics

GraphPad Prism Version 7.0 (GraphPad Software, La Jolla, CA, USA) was used to generate graphical figures and to perform statistical analysis. In MTT assay, non liner regression was used in the statistical study. For MethylScreen assay and qPCR study one-tailed student t test was used in the statistical study. Data are expressed as the mean ± SD. Statistical significance was defined as * = *P* ≤ 0.05, ** = *P* ≤ 0.01, *** = *P* ≤ 0.001.

## Supplementary Information

Supplementary Information 1.

Supplementary Information 2.

Supplementary Information 3.

## Data Availability

All data generated or analyzed during this study are included in the manuscript.

## Abbreviations

EMT: Epithelial mesenchymal transition
5-aza: 5-Azacytidine
MSRE: Methylation sensitive restriction enzymes
MDRE: Methylation dependent restriction enzymes
HM: Hypermethylated DNA fraction
UM: Unmethylated DNA fraction
IM: Intermediately methylated DNA fraction
TSS: Transcription start site
