# Supplementary material for: Investigation of role of CpG methylation in some epithelial mesenchymal transition gene in a chemoresistant ovarian cancer cell line
Source: Sci Rep. 2022 May 6;12:7494. doi: 10.1038/s41598-022-11634-6 (PMC9076839; doi:10.1038/s41598-022-11634-6)
Supplement: Supplementary file 1 — Supplementary Information 1. [file 41598_2022_11634_MOESM1_ESM.pdf]

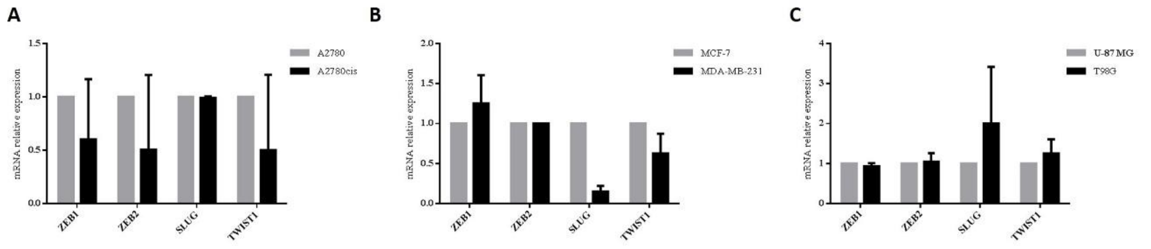

Supplementary (S1):

Resistant cancer cells display molecular changes consistent with partial EMT. Insignificant changes in gene expression of *ZEB1*, *ZEB2*, *SLUG*, *TWIST1* genes were assessed using qPCR, in (A) A2780cis compared to parental cells A2780. (B) MDA-MB-231 cisplatin resistant cells compared to MCF7 cisplatin sensitive cells. (C) T98G cisplatin resistant cells compared to U-87 MG the more sensitive cisplatin cells.
