# Supplementary material for: Investigation of role of CpG methylation in some epithelial mesenchymal transition gene in a chemoresistant ovarian cancer cell line
Source: Sci Rep. 2022 May 6;12:7494. doi: 10.1038/s41598-022-11634-6 (PMC9076839; doi:10.1038/s41598-022-11634-6)
Supplement: Supplementary file 2 — Supplementary Information 2. [file 41598_2022_11634_MOESM2_ESM.pdf]

Supplementary (S2): Primer sequences used for quantitative real-time PCR.

Gene symbol	Primer's sequence (5'-3')
ZEB1	F- CCTGCCAACAGACCAGACAGTGTT
	R- CCCAGGATTTCTTGCCCTTCCTTTC
ZEB2	F- CGGTGCAAGAGGCGCAAACAAG
	R- GGAGGACTCATGGTTGGGCAC
SLUG	F-CAGCGAACTGGACACACATACAG
	R- AGCTGAGGATCTCTGGTTGTGGT
TWIST1	F- TCTTACGAGGAGCTGCAGACGCA
	R- ATCTTGGAGTCCAGCTCGTCGCT
