# Supplementary material for: Investigation of role of CpG methylation in some epithelial mesenchymal transition gene in a chemoresistant ovarian cancer cell line
Source: Sci Rep. 2022 May 6;12:7494. doi: 10.1038/s41598-022-11634-6 (PMC9076839; doi:10.1038/s41598-022-11634-6)
Supplement: Supplementary file 3 — Supplementary Information 3. [file 41598_2022_11634_MOESM3_ESM.pdf]

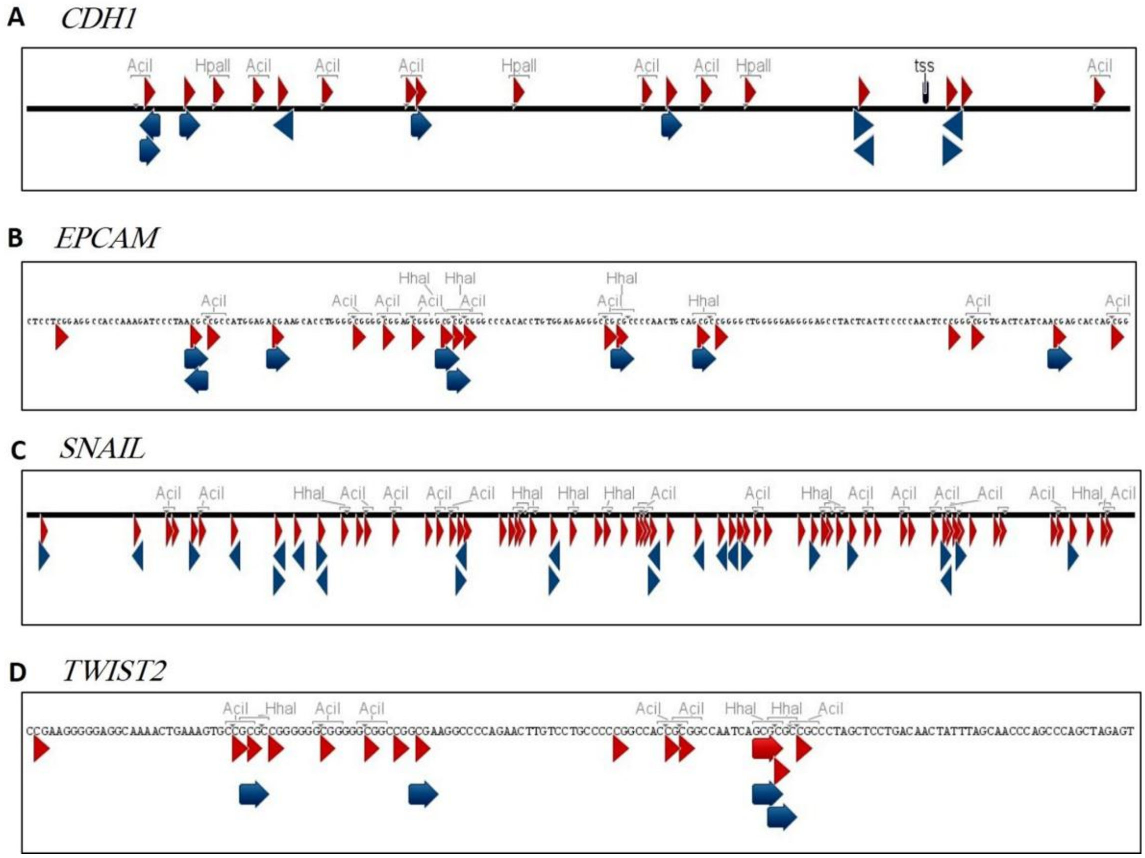

Supplementary (S3):

Illustrations showing the studied section of *CDH1* gene (A), *EPCAM* gene (B), *SNAIL* gene (C) and *TWIST2* gene (D). CpGs dinucleotide are shown as red arrows, restriction sites of methylation-sensitive enzymes MSRE are indicated as vertical domain, restriction sites of methylation-dependent enzymes MDRE are shown as blue arrows.
